# The Impact of Violence on the Anxiety Levels of Healthcare Personnel During the COVID-19 Pandemic

**DOI:** 10.3389/fpsyt.2021.761555

**Published:** 2021-11-05

**Authors:** Mariá Romanio Bitencourt, Lincoln Luís Silva, Ana Carolina Jacinto Alarcão, Amanda de Carvalho Dutra, Marcos Rogério Bitencourt, Giovana Jorge Garcia, Luciano de Andrade, João Ricardo Nickenig Vissoci, Sandra Marisa Pelloso, Maria Dalva de Barros Carvalho

**Affiliations:** ^1^Graduation Program in Health Sciences, State University of Maringá, Maringá, Brazil; ^2^Graduation Program in Biosciences and Physiopathology, State University of Maringá, Maringá, Brazil; ^3^Faculdade Adventista Paranaense, Ivatuba, Brazil; ^4^Department of Medicine, University of Cesumar, Maringá, Brazil; ^5^Duke Global Health Institute, Duke University, Durham, NC, United States

**Keywords:** COVID-19, violence, healthcare personnel, psychological violence, occupational health, anxiety

## Abstract

**Introduction:** The COVID-19 pandemic stressed the importance of healthcare personnel. However, there is evidence of an increase in violence against them, which brings consequences, such as anxiety. The aim of this study was to analyze the anxiety levels of health professionals who have or not suffered violence during the COVID-19 pandemic, and verify the variables associated with the risk of starting to take medication for anxiety.

**Methods:** We assessed the anxiety profile of health professionals in Brazil through an online questionnaire, using the Generalized Anxiety Disorder 7-item Scale (GAD-7), in relation to groups of participants who have or not suffered violence during the COVID-19 pandemic. We used Cronbach's alpha reliability coefficient to check the consistency of the responses, and the effect size using the r coefficient. Principal Component Analysis was used to verify the differences in anxiety scores between the two groups. Logistic regression analysis was also used to verify the variables associated with the risk of starting medication for anxiety and considered statistically significant when *p* < 0.05.

**Results:** A total of 1,166 health professionals participated in the study, in which 34.13% had a normal anxiety profile, 40.14% mild, 15.78% moderate, and 9.95% severe. The mean score of the sum of the GAD-7 was 7.03 (SD 5.20). The group that suffered violence had a higher mean (8.40; SD 5.42) compared to the group that did not (5.70; SD 4.60). In addition, the median between both groups was significantly different (7.0 vs. 5.0; *p* < 0.01). Approximately 18.70% of the participants reported having started taking medication to treat anxiety during the pandemic. The factors that increased the chances of these professionals starting medication for anxiety *p* < 0.05 were having suffered violence during the pandemic (OR 1.97; 95% CI 1.42–2.77), being nurses (OR 1.61; 95% CI 1.04–2.47) or other types of health professionals (OR 1.58; 95% CI 1.04–2.38), and having a mild (OR 2.11; 95% CI 1.37–3.34), moderate (OR 4.05; 95% CI 2.48–6.71) or severe (OR 9.08; 95% CI 5.39–15.6) anxiety level.

**Conclusion:** Brazilian healthcare professionals who have suffered violence during the pandemic have higher anxiety scores and higher risk to start taking anxiety medication.

## Introduction

The novel Coronavirus (COVID-19) pandemic has had a strong impact on the mental health of the population in general. Before the pandemic, the Brazilian prevalence of anxiety disorders was 9.3% (1). However, some studies have revealed an increasement of 7.4-fold during de COVID-19 pandemic in Brazil (2). According to a systematic review including articles from China, Spain, Italy, Iran, US, Turkey, Nepal and Denmark, rates ranged from 6.33 to 50.90% (3).

Healthcare professionals have been particularly impacted, with an increased occurrence of symptoms related to anxiety, depression, insomnia, stress, fear and frustration having been observed in several studies (4–6). The mental health of such professionals is vulnerable to the impacts of the pandemic due to the high number of cases and deaths of patients and coworkers, not to mention the fear of being infected and taking the virus to their homes (7). Other negative factors are precarious working conditions, long shifts and lack of personal protective equipment (PPE) (6, 8).

As of July 2021, Brazil has experienced more than 120 thousand cases of COVID-19 in health professionals, from a total of 20 million cases and 558 thousand deaths in the country (9, 10). Although those professionals in the frontline are seen as heroes by most people, not everyone acknowledges the importance of the role they play (11). There have been reports of attacks against health professionals not only in Brazil, but worldwide, which includes stopping them from entering public places or using public transportation, discrimination, physical violence and insults (12–16). Therefore, the strain on the mental health of these professionals may be further exacerbated as a result of violent verbal or physical outbursts from the general public or patients (8).

Several international studies have been carried out during the pandemic addressing discrimination and violence against healthcare personnel (8, 13, 14), and others on anxiety and mental health (5, 6, 8, 17). However, to date, no studies have been found in Brazil relating anxiety to the violence suffered by healthcare professionals during the COVID-19 pandemic. Therefore, the aim of this study was to analyze the anxiety levels of healthcare professionals who both have and have not personally experienced violence in and outside of the workplace during the COVID-19 pandemic in Brazil. This study also assessed what variables increased the likelihood that a healthcare professional would begin taking medication to manage symptoms of anxiety.

## Materials and Methods

### Study Design

This is a cross-sectional study involving healthcare professionals in Brazil. They answered an online questionnaire containing questions about violence, anxiety and medication use during the COVID-19 pandemic. The questionnaire was available for completion for 15 days from October 1st, 2020.

### Setting

#### Sampling Procedure

People willing to participate were recruited using a snowball sampling method (17). No incentive was provided for the participants to complete the survey because the Ethical Committee does not allow any type of incentive.

#### Eligibility Criteria

The study participants were health professionals who work in Brazil. They were invited through social media to answer an online questionnaire available on Google Forms and share it with other healthcare professionals. Their participation was voluntary and anonymous, and the individuals signed an online consent form before completing the questionnaire.

#### Questionnaire

The questionnaire consisted of 31 questions, of which eight refer to sociodemographic aspects, ten are related to profession and workplace, five are about violence and eight questions about anxiety. Regarding the eight anxiety-related questions, seven of them are the ones that compose the Generalized Anxiety Disorder 7-item Scale (GAD-7), and the last one was whether the participants started to use anxiety medications during the pandemic.

**Figure 1:**
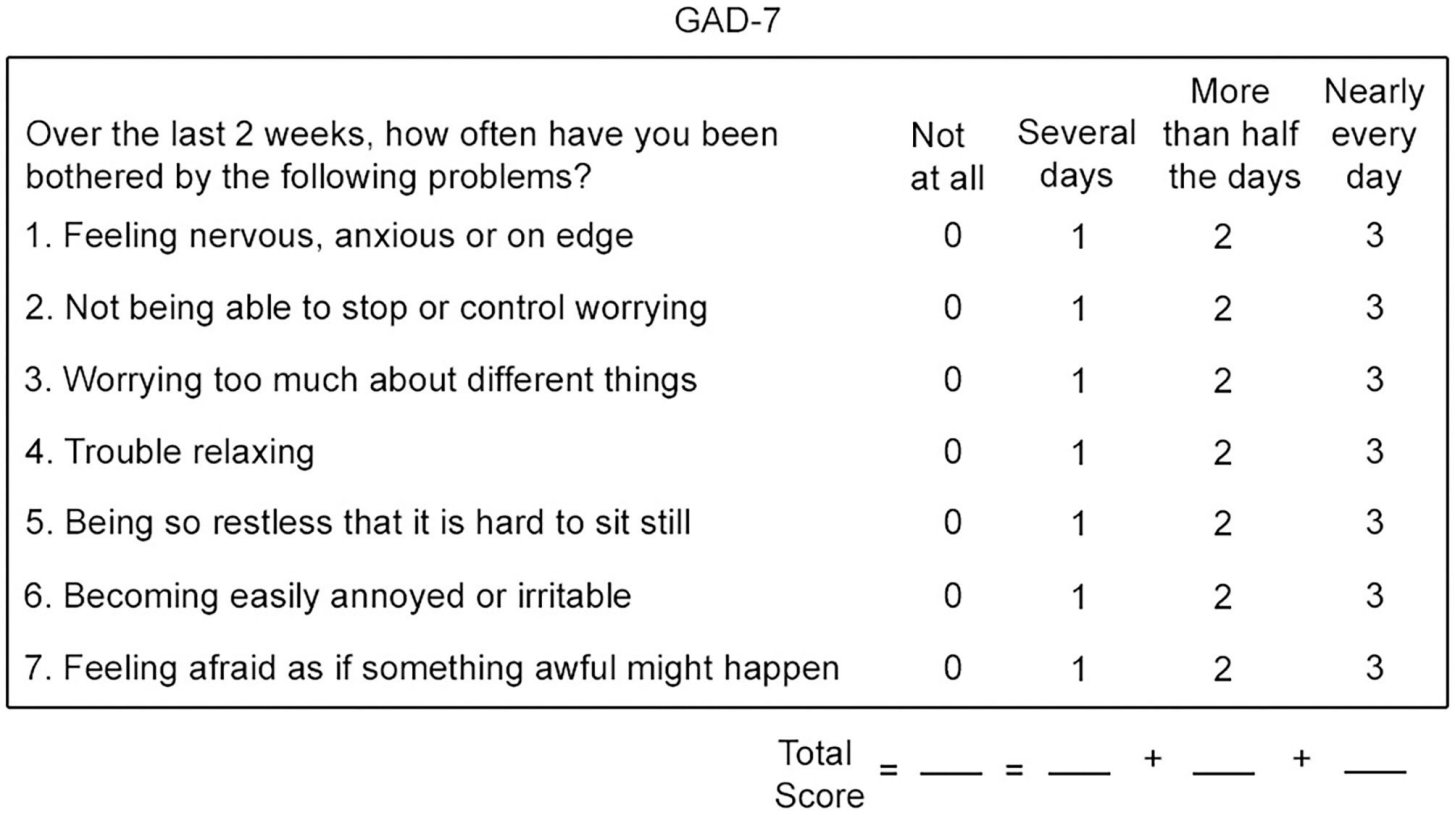
Generalized anxiety disorder 7-item scale (GAD-7).

The GAD-7 is a self-report questionnaire that has been proven to be a reliable and valid measuring tool to assess the symptoms of generalized anxiety in different clinical contexts for the population in general (18, 19). It was created by Spitzer et al. (20) and validated by Kroenke et al. (21) in accordance with the criteria provided by the Diagnostic and Statistical Manual of Mental Disorders. Total scores range from 0 to 21, and the highest ones indicate higher levels of symptom severity. Regarding the severity classifications, we followed the recommendations of the original authors: none/normal (0–4), mild (5–9), moderate (10–14), and severe (more than 15) anxiety. Figure 1 shows the GAD-7 questions and scores.

### Data Analysis

#### Source

The data obtained were organized on an Excel® spreadsheet (Microsoft Office, Microsoft Corporation, USA) and are available on an online repository on http://doi.org/10.6084/m9.figshare.c.5585541.

#### Sociodemographic Characteristics

The sociodemographic characteristics of the studied population was published elsewhere in a previous paper (22).

#### Analysis of Differences Between the Groups

The study population was divided into two groups: those who have suffered violence during the pandemic, and those who have not. We compared their anxiety levels and the variables associated with the risk of starting medication for anxiety. For the analysis of descriptive statistics, we used absolute and relative frequencies, means, and standard deviation.

The reliability coefficient (Cronbach's alpha) was used to verify the consistency of the participants' responses. Then, the effect size (*r*) of the average score item was analyzed to verify the difference comparing the answers provided by each group (23). The normality test was performed beforehand, as required for performing the Mann-Whitney test in order to verify whether the difference between groups exists.

Finally, principal component analysis (PCA) with two factors and varimax rotation was performed, since previous studies also detected two-dimensional structures to verify the most correlated GAD-7 variables (>0.6) with variability of scores for anxiety (24).

#### Analysis of Variables Associated With the Beginning of Using Medication for Anxiety

We performed a univariate logistic regression analysis of the study population using the following variables: sex, age range, race, education level, having children, having a partner, profession, length of service/experience availability of PPE at the workplace, if the PPE is considered adequate, weekly workload working hours, monthly income, having had COVID-19, being a frontline health professional, having suffered violence before the pandemic, having suffered violence during the pandemic, family members having suffered violence for being close to healthcare professionals, and, finally, the severity level within the anxiety scale.

Use of medication as a dependent variable was considered in order to estimate the risk of starting medication use for anxiety during the pandemic. Those variables with *p*=0.1 were selected to be analyzed together in the multivariate logistic regression analysis, and considered statistically significant when *p* < 0.05.

All statistical analyses were carried out with the software R Studio 1.1.456 using car, psych, corrplot and ggplot2 packages.

### Ethics

The study was approved by the Ethics Committee of the State University of Maringá, under registration number 37712820.4.0000.0104, in accordance with Normative Resolution 510/2016.

## Result

### Reliability Analysis of the Responses

The reliability coefficient (Cronbach's alpha) of the GAD-7 for the study population was 0.91, which means that the answers provided by the participants were considered highly reliable. That enabled us to carry out the other analyses regarding the population.

### Participant's Characteristics

The sociodemographic profile and anxiety level of the 1,166 participants are described in Table 1. As for the classification of their anxiety level, about 398 (34.13%) had a normal level, 468 (40.14%) mild, 184 (15.78%) moderate, and 116 (9.95%) severe.

**Table 1:** Sociodemographic and professional characteristics of the participants according to their anxiety level.

**Characteristics**	**Classification**								**Total**	
	**Normal**		**Mild**		**Moderate**		**Severe**			
	***N***	**%**	***N***	**%**	***N***	**%**	***N***	**%**	***N***	**%**
	**398**	**34.13**	**468**	**40.14**	**184**	**15.78**	**116**	**9.95**	**1,166**	**100**
**Sex**										
Male	132	11.32	104	8.92	33	2.83	19	1.63	288	24.70
Female	266	22.81	364	31.22	151	12.95	97	8.32	878	75.30
**Age range**										
18–39	217	18.61	280	24.01	133	11.41	89	7.63	719	61.66
40–59	151	12.95	167	14.32	48	4.12	27	2.32	393	33.70
60–80	29	2.49	21	1.80	3	0.26	0	0.00	53	4.55
>80	1	0.09	0	0.00	0	0.00	0	0.00	1	0.09
**Race**										
White	332	28.47	375	32.16	140	12.01	82	7.03	929	79.67
Black	8	0.69	7	0.60	3	0.26	2	0.17	20	1.72
Others	58	4.97	86	7.38	41	3.52	32	2.74	217	18.61
**Education level**										
No academic degree	43	3.69	48	4.12	26	2.23	20	1.72	137	11.75
Undergraduate degree	42	3.60	55	4.72	35	3.00	26	2.23	158	13.55
Residency	247	21.18	286	24.53	105	9.01	56	4.80	694	59.52
Post-graduation degree	66	5.66	79	6.78	18	1.54	14	1.20	177	15.18
**Having children**										
No	155	13.29	201	17.24	94	8.06	64	5.49	514	44.08
Yes	243	20.84	267	22.90	90	7.72	52	4.46	652	55.92
**Partner**										
No partner	125	10.72	156	13.38	78	6.69	50	4.29	409	35.08
With a partner	273	23.41	312	26.76	106	9.09	66	5.66	757	64.92
**Profession**										
Physician	232	19.90	264	22.64	96	8.23	49	4.20	641	54.97
Nurse	58	4.97	76	6.52	26	2.23	20	1.72	180	15.44
Nursing assistant/technician	36	3.09	43	3.69	26	2.23	24	2.06	129	11.06
Others^*^	72	6.17	85	7.29	36	3.09	23	1.97	216	18.52
**Length of service/experience (years)**										
≤5	82	7.03	113	9.69	69	5.92	43	3.69	307	26.33
6–10	100	8.58	123	10.55	52	4.46	38	3.26	313	26.84
11–20	112	9.61	139	11.92	47	4.03	28	2.40	326	27.96
>20	104	8.92	93	7.98	16	1.37	7	0.60	220	18.87
**Weekly workload (hours)**										
≤36	106	9.09	125	10.72	42	3.60	27	2.32	300	25.73
37–44	134	11.49	159	13.64	56	4.80	37	3.17	386	33.10
>44	158	13.55	184	15.78	86	7.38	52	4.46	480	41.17
Monthly income (Brazilian Reals)^**^										
≤5 thousand	123	10.55	156	13.38	79	6.78	60	5.15	418	35.85
5–10 thousand	65	5.57	81	6.95	40	3.43	19	1.63	205	17.58
11–20 thousand	102	8.75	132	11.32	35	3.00	26	2.23	295	25.30
>20 thousand	108	9.26	99	8.49	30	2.57	11	0.94	248	21.27
**Had COVID-19**										
No	350	30.02	412	35.33	162	13.89	98	8.40	1,022	87.65
Yes	48	4.12	56	4.80	22	1.89	18	1.54	144	12.35
**PPE at workplace**										
No	43	3.69	79	6.78	31	2.66	32	2.74	185	15.87
Yes	355	30.45	389	33.36	153	13.12	84	7.20	981	84.13
**PPE adequate**										
No	88	7.55	166	14.24	81	6.95	53	4.55	388	33.28
Yes	310	26.59	302	25.90	103	8.83	63	5.40	778	66.72
**Frontline health professional**										
No	183	15.69	218	18.70	62	5.32	48	4.12	511	43.83
Yes	215	18.44	250	21.44	122	10.46	68	5.83	655	56.17
**Violence before the pandemic**										
No	350	30.02	371	31.82	140	12.01	85	7.29	946	81.13
Yes	48	4.12	97	8.32	44	3.77	31	2.66	220	18.87
**Family members suffered violence**										
No	347	29.76	377	32.33	130	11.15	82	7.03	936	80.27
Yes	51	4.37	91	7.80	54	4.63	34	2.92	230	19.73
**Violence during the pandemic**										
No	259	22.21	233	19.98	68	5.83	32	2.74	592	50.77
Yes	139	11.92	235	20.15	116	9.95	84	7.20	574	49.23
**Start medication during pandemic**										
No	367	31.48	391	33.53	131	11.23	59	5.06	948	81.30
Yes	31	2.66	77	6.60	53	4.55	57	4.89	218	18.70

* *Other healthcare professionals: speech therapists, physiotherapists, psychologists, social work assistants, nutritionists, odontologists, pharmacists/biochemists, occupational therapists, communitarian health agents, health technicians (laboratories, radiology and other types of imaging exams), and other members of healthcare staff (rescue workers, ambulance drivers and stretcher bearers)*.

** *US$1 = 5.40 Brazilian Reals (according to the dollar exchange rate on August 19th, 2021)*.

The variables that have the greatest proportion comparing mild, moderate or severe anxiety levels to normal levels, were as follows: women (69.70%), aged 18–39 (69.81%), professionals who hold a graduate degree (73.41%), those who have no children (69.84%), those who do not have a partner (70.17%), nursing technicians or assistants (72.09%), those who had been working for <5 years in healthcare (73.28%), those who work over 44 h a week (67.08%), those whose income is inferior to 5 thousand Brazilian Reals (70.57%), those who had been diagnosed with COVID-19 (66.67%), those who had no access to PPE at their workplace (76.75%) or had access to inadequate PPE (77.31%), frontline healthcare professionals (67.17%), those who had suffered violence before (78.18%) and during the pandemic (75.18%) and, finally, those who started taking medication for anxiety during the pandemic (85.77%).

### Populational Differences

**Figure 2:**
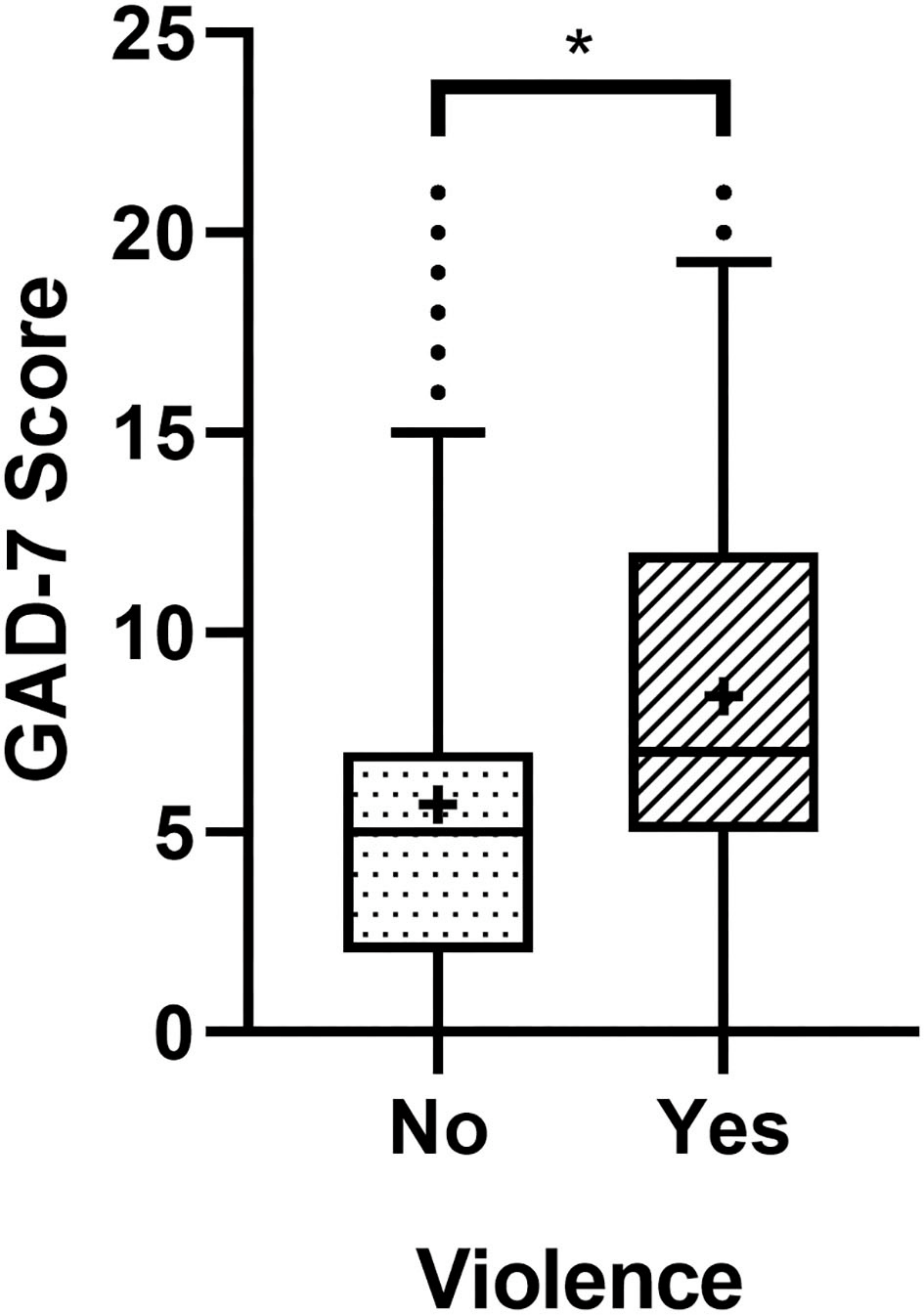
Boxplots showing the GAD-7 scores of the participants who have or not suffered violence during the pandemic. The dots above the superior limit represent the outliers. The crosses inside the boxplots indicate the score average of their respective group, and * indicates difference between the groups (*p* < 0.01).

The study population was divided into 2 groups: (1) those who have suffered violence during the pandemic and (2) those who have not. We used the Mann-Whitney Test, which showed that the median of the score for those who were victims of violence is different from that of the group that suffered no violence (W = 118,187, *p* < 0.01) (7.0 vs. 5.0). Dispersion of the scores of both groups can be seen in Figure 2.

Regarding the score of the GAD-7 items shown in Table 2, the mean score for the group that did not suffer violence ranged from 0.42 to 0.99, while the range of the group that suffered violence was between 0.75 and 1.42. Furthermore, the differences between both groups, expressed in terms of effect sizes, were found for items 1–6, which were those with the greatest divergences (*r* > 0.21). This means that items 1–6 had a slight increase in the average effect regarding the group that suffered violence.

**Table 2:** Mean, standard deviation, and *r* coefficient differences between groups.

**GAD7**	**Group**				**General**		
	**No violence**		**Yes violence**				
	**Mean**	**SD**	**Mean**	**SD**	**Mean**	**SD**	***r***
1	0.99	0.84	1.39	0.95	0.83	0.87	**0.22**
2	0.74	0.80	1.15	0.99	0.80	0.77	**0.21**
3	0.99	0.87	1.42	1.00	1.02	0.94	**0.22**
4	0.97	0.88	1.36	0.95	1.29	0.92	**0.21**
5	0.42	0.70	0.75	0.88	1.09	1.03	**0.21**
6	0.89	0.85	1.30	0.96	0.66	0.86	**0.21**
7	0.70	0.82	1.03	0.99	0.87	0.89	0.17
Sum score	5.70	4.60	8.40	5.42	7.03	5.20	**0.26**

*In bold: Higher difference between groups*.

*SD, Standard deviation; r, Size effect*.

In general, the means of the items were higher for the group that suffered violence. Considering the sum total of the group that suffered violence, the mean was 8.40, with a standard deviation (SD) of 5.42, while the group that did not suffer violence had an average of 5.70 and a SD of 4.60. As we can see, the descriptive parameters of the scores for anxiety are higher amongst the population that suffered violence.

### Variability of the Answers

The analysis of the main components of the population that did not suffer violence showed that two dimensions account for 75% of the data variance. The highest correlations in the first dimension (>0.6) were obtained between items 1- Nervous, anxious, on edge; 2- Not being able to stop or control worrying; 3- worrying too much about different things; 4- Trouble relaxing, and 6- Becoming easily annoyed or irritable. As for the second dimension, the items that stood out were 5- Being so restless that it is hard to sit still, and 7- Feeling afraid as if something awful might happen (Table 3).

**Table 3:** Principal component analysis of each item in GAD-7 of the population study.

**GAD-7**	**Violence during pandemic COVID-19**			
	**No**		**Yes**	
	**Dimension 1**	**Dimension 2**	**Dimension 1**	**Dimension 2**
1	**0.845**	0.274	**0.842**	0.224
2	**0.865**	0.256	**0.811**	0.317
3	**0.836**	0.346	**0.707**	0.487
4	**0.790**	0.377	**0.782**	0.287
5	0.404	**0.668**	**0.639**	0.366
6	**0.669**	0.423	**0.830**	0.143
7	0.229	**0.878**	0.246	**0.947**
Loadings	3.448	1.799	3.637	1.520
Proportion variance	0.493	0.257	0.520	0.217
Cumulative variance	0.493	0.750	0.520	0.737

*In bold: High correlations (>0.6)*.

The population that suffered violence, on the other hand, also performed better within two dimensions that account for 73.7% of the data variance. The highest correlations (>0.6) in the first dimension refers to the following variables: 1–6 for the first dimension, and 7 as for the second dimension (Table 3).

This analysis showed that the items of the group that suffered violence have different information variability compared to the group that did not. In addition, item 5 had the most discrepant correlation between the two groups.

**Figure 3:**
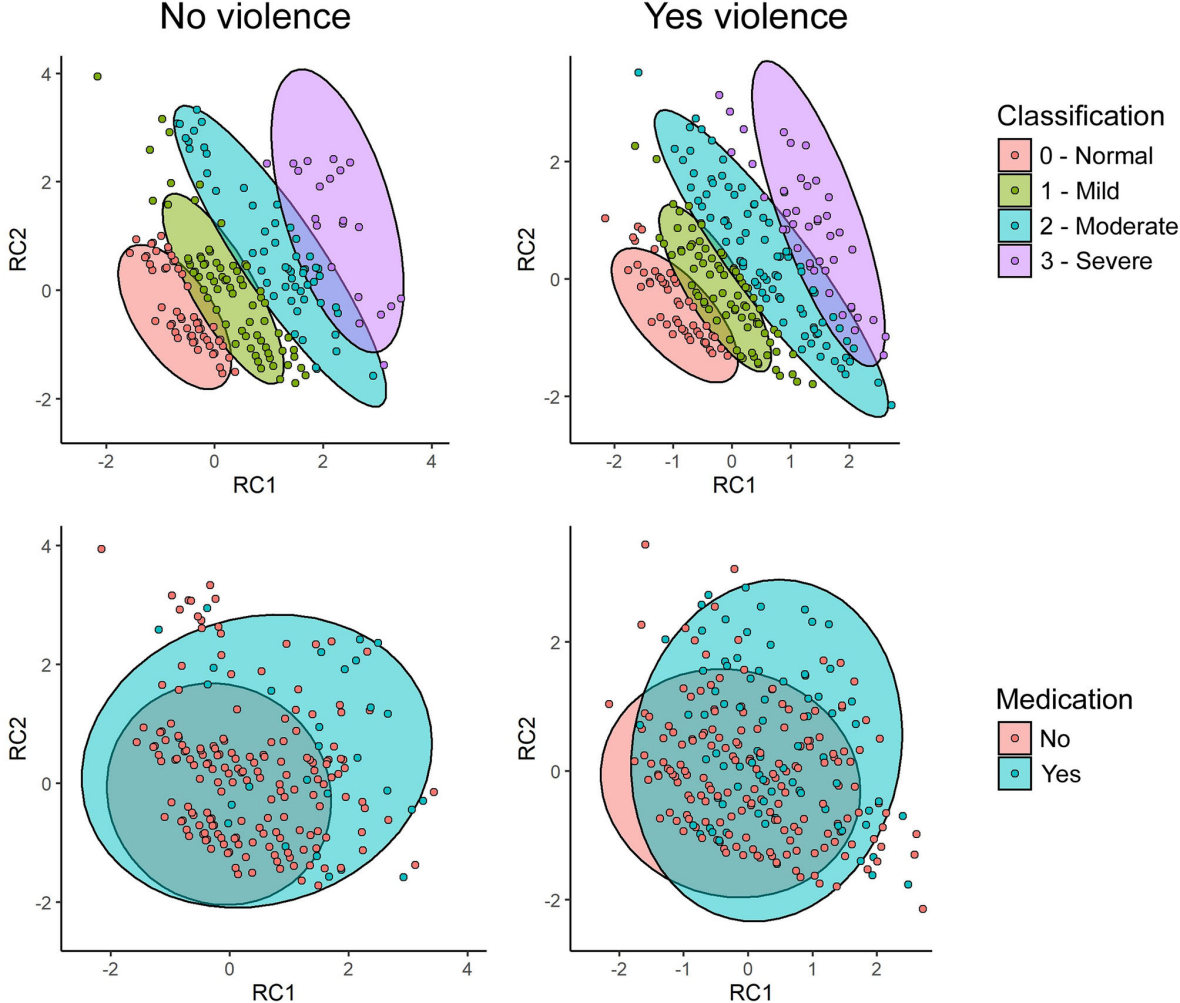
Distribution of the participants' anxiety scores, and grouping related to severity classification and use of medication between the group that suffered violence and the one that did not.

Figure 3 shows the dispersion of the scores of both groups in space. Each dot represents a participant's score. The first row contains the ellipses with the score distributions regarding the severity classification of the GAD-7 scale. The second row refers to medication use. Dots in the lower left quadrant of each figure represent the lowest scores, while the upper right quadrant shows the highest scores. The differences in color of the dots and ellipses represent the category that the dots and ellipses belong to. Dots outside the ellipses are considered outliers. Therefore, when looking at the first row, it is possible to see an ordered scale of score classification in the two groups regarding the anxiety level as normal (0), mild (1), moderate (2), and severe (3). In addition, the second row indicates that there are participants with similar scores among themselves. However, they belong to different groups when it comes to use of medication. Thus, it is suggested that, besides violence, there may be other factors involved in the use of medication by the participants to treat anxiety.

### Involved Variables That Increase the Chances of Using Medication

Considering the aforementioned variables, having suffered violence during the pandemic increases patients' chances of starting to use anxiety medication by 97%. Moreover, being a nurse, nursing assistant or technician, or having other healthcare-related jobs (not doctors) increase the chances by, 61%, 18%, and 58%, respectively. Finally, anxiety levels classified as mild, moderate, or severe increase the odds by 111%, 305%, and 808%, respectively (Table 4).

**Table 4:** Multivariate analysis of variables related to medication initiation during the pandemic.

**Variables**	**OR**	**95% CI**	***p*-value**
**Violence during the pandemic**			
No	–	–	–
Yes	1.97	1.42–2.77	<0.01
**Classification**			
Normal	–	–	–
Mild	2.11	1.37–3.34	<0.01
Moderate	4.05	2.48–6.71	<0.01
Severe	9.08	5.39–15.6	<0.01
**Profession**			
Physician	–	–	–
Nurse	1.61	1.04–2.47	0.03
Nursing assistant/technician	1.18	0.71–1.92	0.51
Others	1.58	1.04–2.38	0.03

*OR, Odds ratio; 95% CI, 95% confidence interval*.

## Discussion

In this work, healthcare professionals in Brazil who suffered any violence during the COVID-19 pandemic presented higher scores of anxiety in comparison to those who did not. Almost half of the participants (49.23%) reported having suffered some type of violence during the pandemic, and 30% of that group had not been victims of violence before the pandemic this reflects what has been observed in other studies. Corroborating our study, another one carried out with Chinese health professionals also showed that those who suffered violence in the workplace had a lower quality of life (25). In addition, a study in Saudi Arabia demonstrated the risk factors associated with higher levels of anxiety: being a nurse, having a previous history of anxiety, having a chronic disease and being a smoker (26).

Globally, many professionals were victims of violence in their workplace by family members of hospitalized patients who happened to be distressed by the uncertain results of effective therapies against COVID-19, risk of death and suspension of hospital visits (14, 25). Violence also occurs out of the professionals' workplace due to fear of being infected, since they work in direct contact with COVID patients (27). That leads people (even family and friends) to avoid or socially reject healthcare personnel (28).

Anxiety has been the most common mental health-related symptom presented by health professionals during the COVID-19 pandemic (17). According to the WHO, Brazil already was the country with the highest rate of people suffering from anxiety disorders in the world (1, 29). The data provided by the WHO informs that 9.3% of the Brazilian population have an anxiety disorder (1). There is no data informing the prevalence of anxiety disorder in Brazil during the pandemic to date. However, studies have shown a prevalence of anxiety among health professionals about 40% in others countries (19). Therefore, it is expected to verify an increased amount of people suffering from anxiety disorders in a near future in Brazil.

In this study, 75.3% of the participants were women and 52.4% were classified as having some level of anxiety. Data have showed that 70% of frontline professionals are women who, in addition to work, have to meet the demands of home, caring for children and other family members (30). This is in consonance with studies that linked anxiety, stress, burnout, and depression to being a female, at a young age, working as a nurse, and directly dealing with patients with COVID-19 (31). This overload and the pressure of having to fulfill all these roles can lead to feelings of frustration, anxiety and stress (8).

In fact, the overall prevalence of mental health diseases in women is higher, compared to men (32). However, men are far less likely to seek mental health treatment due to stigma (14, 32), according systematic review, in which showed that women have less stigma regarding seeking help for mental illness (33). Generally, social norms and cultural expectations enhance a powerful and dominant figure of men, making it difficult for them to express negative emotions and seek mental health care when necessary (32). The stigma related to mental illness can involve factors such as social, personal, professional, and cultural stigma. All these aspects act as barriers for the sick person and make it difficult to seek help.

Apart from women, nursing professionals are more likely than physicians to suffer from stress (8). Studies involving health professionals during the pandemic showed that the longer the time in contact with patients and their relatives, and the closer these professionals are to them, the greater is the number of mental health-related symptoms presented by nurses (26, 34). In this study, working as a nurse increased the chances of an individual starting to take anxiety medication by 61%. Nevertheless, reluctance in seek mental health treatment among physicians is common due to stigma. Physicians fear that if they disclose illness to their colleagues they will be judged as weak and less capable of doing their jobs, and maybe our findings could be due to this (35).

When asked about the use of medication to treat anxiety, 18.70% of the participants reported to have started taking it during the pandemic. Another study has shown that healthcare professionals with high levels of anxiety before the pandemic were more likely to seek help from mental health professionals (26). Yet, many of them do not admit that they need help, since their role as health professionals is to treat sick people. Thus, they are slow to seek help, or end up self-medicating (36). Self-medication among health professionals has been the target of other studies showing that many professionals start taking medication on their own since they have easy access to it, and because they can get prescriptions from co-workers (36, 37).

The COVID-19 pandemic had consequences that go beyond the disease and the number of deaths caused by it, for example: increasement of mental health disorders mostly anxiety and depression, financial problems for those who lost their jobs, feelings of guilt due to the loss of family members and social isolation (26, 38–42). Studies on public health crises emphasize that mental health care should be as paramount as primary care (42). Therefore, government officials must immediately pay attention to the effects of the pandemic in order to prevent further damage to the mental health of these professionals, whose work is crucial in the fight against COVID-19, but also because they need to be mentally well to play their roles in society.

## Limitations

The limitation faced by this research is the fact that the sampling was performed without a probabilistic sampling in Brazil, meaning that the number of participants is not a representative sample. In addition, snowball sampling can create an imbalanced number of participant classes, so participants from some groups may have far greater numbers than others. However, since Brazil is a continental-size country, this work counted on the participation of people from all regions, which allowed a broader view of the violence issue in the pandemic scenario.

The GAD-7 scale is a screening tool for anxiety, and the cross-sectional nature of this study does not allow for a proven diagnosis, which must be carried out by experts. Anyhow, the aim of this study was to verify the degree of anxiety based on the participants' responses.

## Conclusion

In Brazil, health professionals who have suffered violence during the COVID-19 pandemic have higher anxiety scores in comparison to those who have not. Additionally, being nurses or other types of health professionals, and having a mild, moderate, or severe anxiety level have higher risk to start taking anxiety medication.

## Data Availability Statement

The datasets for this study can be found in the Figshare repository: http://doi.org/10.6084/m9.figshare.c.5585541.

## Ethics Statement

The studies involving human participants were reviewed and approved by Comite de Ética Universidade Estadual de Maringá. The patients/participants provided their written informed consent to participate in this study.

## Author Contributions

MariB conceived the study, drafted the manuscript, and collected the data. LS and LA conducted the statistical analysis. AA designed the study. GG, AD, MarcB, JN, and SP helped to revise the manuscript. MC supervised the study. All authors have read and approved the final manuscript.

## Conflict of Interest

The authors declare that the research was conducted in the absence of any commercial or financial relationships that could be construed as a potential conflict of interest.

## Publisher's Note

All claims expressed in this article are solely those of the authors and do not necessarily represent those of their affiliated organizations, or those of the publisher, the editors and the reviewers. Any product that may be evaluated in this article, or claim that may be made by its manufacturer, is not guaranteed or endorsed by the publisher.
